# The effect of coronary slow flow on left atrial structure and function

**DOI:** 10.1038/s41598-021-87193-z

**Published:** 2021-04-05

**Authors:** Zhiyuan Shui, Yunzhi Wang, Mingxue Sun, Yiqun Gao, Shunji Liang, Yiran Wang, Xiaomei Wang, Qin Yu, Shulong Zhang, Li Liu

**Affiliations:** 1 Department of Cardiology, Affiliated Zhongshan Hospital of Dalian University, Dalian, 620061 Liaoning Province China; 2 Department of Cardiology, The Sanmenxia Central Hospital, Sanmenxia, Henan Province China; 3 Department of Cardiology, The People’s Hospital of Sanya City, Sanya, Hainan Province China

**Keywords:** Cardiology, Diseases

## Abstract

The coronary slow flow phenomenon (CSFP) is common in coronary angiography, however its impact on left atrial (LA) function is still controversial. This study aims to evaluate the LA structure and function of patients with CSFP using two-dimensional speckle tracking echocardiography (2D-STE). Consecutive patients scheduled for coronary angiography from January 2016 to September 2017 were enrolled in this study. Patients’ demographic data, clinical histories, laboratory and angiographic findings were collected and recorded. Diagnostic criteria for CSFP is based on Beltrame et al. proposed in 2012. Meanwhile 139 patients who have no significant stenosis (≤ 40%) and normal blood flow were selected as control. All patients received an echocardiographic examination 24 h before coronary angiography. LA structure and function were measured with echocardiography and 2D-STE. Our results showed that among the 1,954 patients who had received coronary angiography, 512 patients were included in the analysis after the exclusion criteria was implemented. Of those, 101 patients met the CSFP criteria (5.5%). CSFP is mainly seen in LAD (~ 70%). There was no statistical difference in baseline characteristics between the CSFP group and control group, except for a higher proportion of smokers in the CSFP group (P = 0.001). The percentage of monocytes is an independent risk factor for the occurrence of CSFP (P = 0.036) after binary logistic regression analysis. The LA global longitudinal strain (LA-GLS, represents reservoir functions) decreased and LA strain rate at late diastole (LA-SRa, represents booster function) increased in patients with CSFP compared to the control group (P < 0.05). Correlation test of continuous variables by Pearson test suggested that LA-GLS was negatively correlated with TIMI frame count (TFC). We concluded that the percentage of monocytes is an independent risk factor for the CSFP; the LA reservoir and booster functions were impaired in patients with CSFP; LA-GLS is negatively correlated with TFC.

## Introduction

Since the coronary slow flow phenomenon (CSFP) was firstly described by Tambe^1^, it has attracted significant attention among cardiologists. The CSFP is diagnosed based on the results of coronary angiography. The current adapted diagnostic criteria was proposed by Beltrame^2^ where CSFP is characterized by delayed distal vessel opacification of contrast in the absence of significant epicardial coronary stenosis (coronary artery stenosis ≤ 40%), with the Thrombolysis in Myocardial Infarction (TIMI) blood flow grade of 2 or corrected TIMI frame count (cTFC) of greater than 27 frames in one or more epicardial vessels. CSFP is still a "phenomenon" because its definition is based on visual findings of delayed contrast filling of the distal coronary artery in the absence of significantly obstructive vascular disease. Its pathophysiological mechanism remains unclear. Some researchers believe it represents a pathology related to an underlying dysfunction of microvascular resistance, thrombocyte dysfunction, oxidative stress, systemic/local inflammation, and/or a combination of all listed factors^3–5^.

CSFP is not rare, with an incidence of approximately 1–5.5% in patients scheduled for coronary angiography^6–8^. Some cardiologists believe it is often underrecognized, and the incidence could be as high as 34%^9^. The clinical manifestations of patients with CSFP are diverse, but more than 80% of patients can have repeated chest pain episodes that could last for an extended period of time^10^ and could also occur at rest^11^. The clinical symptoms range from severe, such as acute coronary syndrome, or mild, such as chest tightness, and sometimes CSF can even cause fatal ventricular arrhythmias^12–14^. Studies have shown that younger patients are more likely to develop CSFP, and the prevalence is higher in men than women^7^. CSFP is more likely seen in smokers^13^, patients with metabolic syndrome and high BMI^15^, and patients with anxiety, depression^16^ and sleep apnea^17^.

CSFP can cause left ventricular dysfunction, mainly left ventricular diastolic dysfunction^18,19^. However, the effect of CSFP on left atrial function has been minimally investigated. A few studies can be found in the existing literature, but the conclusion is controversial^20,21^. The left atrium serves an integral role in cardiac performance and is closely related to left ventricle function. Hemodynamic changes in the left atrium promote the formation of thrombosis, causing thromboembolism, stroke, and atrial fibrillation^22,23^. Abnormal left atrial function can affect left ventricular filling and cause a decrease in cardiac output. Accurate evaluation of the structure and function of the left atrium has important significance in clinic settings^22,24^. Traditional methods using two-dimensional (2D) echocardiography for left atrial function assessment gives limited information depending on hemodynamic loading and geometric measurements^25^ and may not fully reflect the left atrial function. 2D speckle tracking echocardiography (2D-STE) is a relatively new echocardiographic technique developed for quantitative evaluation of cardiac function. This technology quantifies the movement of the myocardium by tracking the trajectory of the myocardial acoustic spot during the cardiac cycle. Because it is a more sensitive technique for the evaluation of cardiac function versus traditional echocardiography, it could provide greater accuracy when determining LA function in CSFP patients^25,26^. In this study we used 2D-STE for evaluating the LA deformation in patients with CSFP and compared the findings with control patients to investigate the effect of CSFP on LA structure and function.

## Patients and methods

### Patients

All consecutive patients scheduled for coronary angiography from January 2016 to September 2017 in the Heart Center of affiliated Zhongshan Hospital of Dalian University were enrolled. The following patients were excluded if they have (1) myocardial infarction, (2) a history of cardiac surgery or percutaneous coronary intervention, (3) severe uncontrollable hypertension or left ventricular hypertrophy, (4) severe liver and kidney dysfunction, (5) heart valve disease, (6) cardiomyopathy, (7) severe infection, (8) malignant tumor, (9) cardiac insufficiency NYHA grade II or above, (10) coronary artery spasm and dilation, (11) myocardial bridge, (12) anemia, (13) thyroid disease, (14) the left bundle branch block or paced rhythm, (15) atrial fibrillation, (16) poor coronary angiography or cardiac ultrasound images. Concurrently, 139 control patients were randomly selected from the Coronary Angiography Data Bank of our hospital. These control patients had insignificant or no coronary stenosis (no more than 40%) and normal blood flow determined by coronary angiography.

Patients’ general clinical information including gender, age, height, weight, BMI, blood pressure, heart rate, as well as laboratory test results (e.g. blood routine, liver and kidney function, high-sensitivity C-reactive protein, homocysteine, and so on), were collected within 3 days before angiography was performed. All patients underwent routine echocardiography within 24 h before coronary angiography.

Written informed consent was obtained from all patients before enrollment. The study protocol was approved by the Dalian University Ethics Committee and was conducted in accordance with the ethical guidelines of the 1975 Declaration of Helsinki.

### Coronary angiography and diagnosis of CSFP

Coronary angiography was performed using the GE Medical System (USA). A catheter was introduced through either the right or left radial artery in all patients and multi-positional images were acquired to fully visualize every segment of the right and left coronary arteries. Iohexol or iopamidol was used as the contrast agent. The diagnostic criteria for CSFP is based on the recommendations of Beltrame JF proposed in 2012^2^. Briefly, there was no coronary artery stenosis or stenosis ≤ 40% at coronary angiography and the delay of distal angiographic contrast agent filling reaches corrected TIMI frame count (cTFC) value > 27 frames (30 frames/s), at least one coronary artery is involved.

### Two-dimensional speckle tracking echocardiography (2D-STE)

Echocardiography was performed on all patients within 24 h before coronary angiography using the Phillips Color Doppler Ultrasound Scanner (Phillips Medical System EPIC 7C, S5-1 probe). The optimal frame rate for 2D image acquisition was set between 60 and 100 frames per second, and images are continuously acquired for three cardiac cycles. All acquired images and cardiac structure measurement methods are based on the 2016 Chinese Adult Echocardiography Examination Measurement Guidelines^27^. The inner diameter of the left atrium was measured at the end of the ventricular systole, including the antero-posterior diameter (LA-ap), the long diameter (LA-l), and the transverse diameter of the left atrium (LA-t). The mean LA diameter was calculated as the average value of the left atrial inner diameter (mLAD), mLAD = (LA-ap + LA-l + LA-t)/3, and the left atrial diameter index (LADI),is calculated as LADI = mLAD/ body surface area (BSA). The two-plane Simpson method was used to calculate the left atrial volume and ejection fraction. The maximum and minimum volumes of the left atrium were corrected with the BSA to obtain the left atrial maximal volume index (LAVI-max), LAVI-max = LAV-max/BSA, and the left atrial minimum volume index (LAVI-min), LAVI-min = LAV-min/BSA. All LA sizes and volume measurements were the average of three different cardiac cycles.

**Figure 1:**
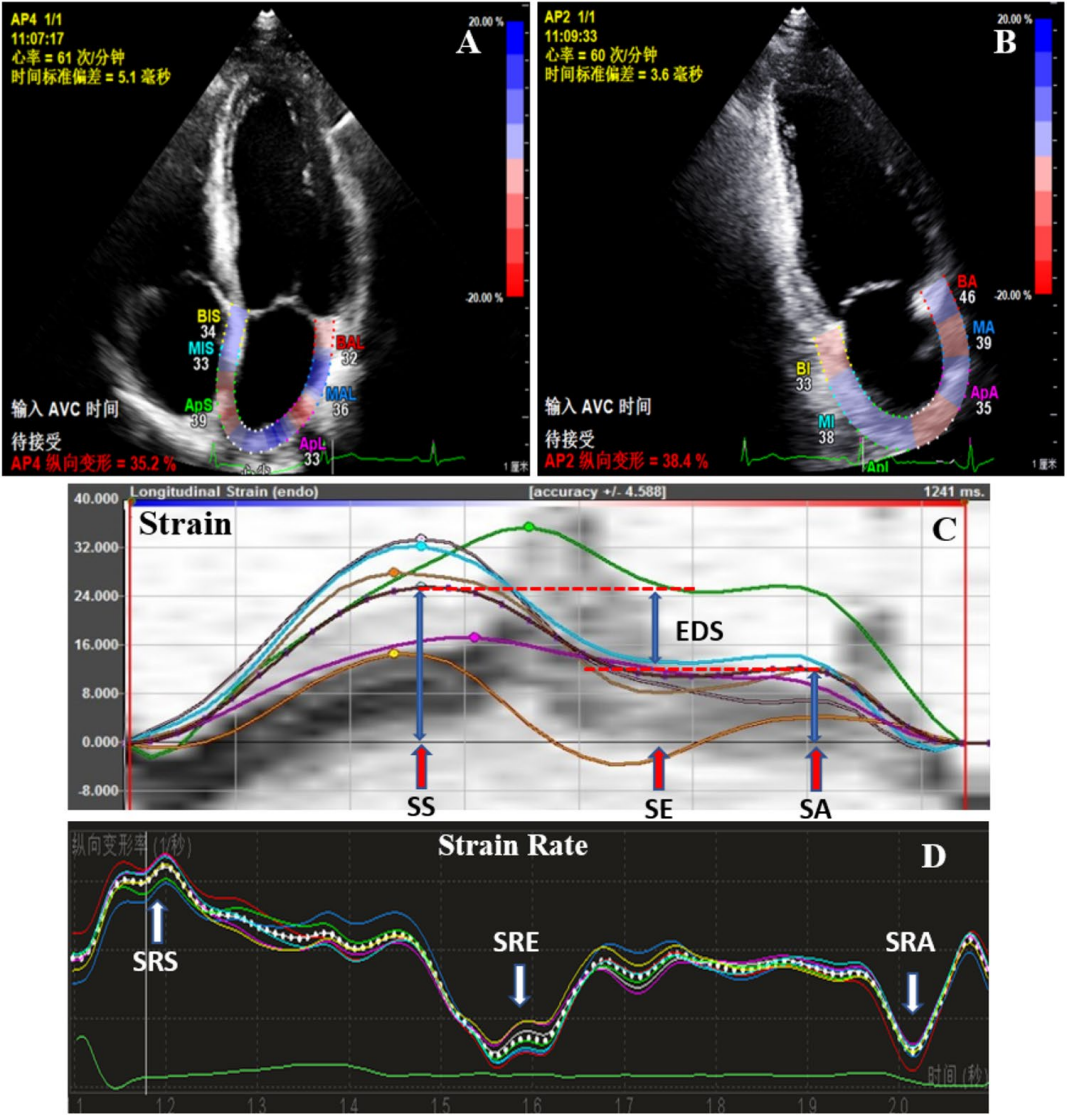
(**A**) Apical 4 chamber view; (**B**) Apical 2 chamber view; (**C**) Strain curve: The longitudinal LA strain curve had a positive systolic peak (SS), an early diastolic plateau (SE), and a late diastolic peak (SA); (**D**) Strain rate curve: the longitudinal LA strain rate curve had a positive systolic peak (SRS), an early negative peak at early diastole (SRE), and a late negative peak at late diastole (SRA). SS and SRS represented the LA reservoir function, EDS (= SS − SA) and SRE reflected the LA conduit, and SA and SRA were the indices of the LA booster function.

For 2D-STE, echocardiographic images were stored at 60–100 frames per second in three cardiac cycles. The strain and strain rate were measured at apical 4- (AP4) and 2-chamber (AP2) views respectively using Qlab10.5 workstation. Initially, the endocardial border of the LA was manually traced in aCMQ mode at end ventricular systole, the epicardial borders of the LA wall were automatically defined through software, and the width was manually adjusted so that the tracking area covered the full thickness of the myocardium. After the adjustment and confirmation of these traced borders, the software automatically calculated and displayed the results (Fig. 1). In patients with adequate image quality, six segments were analyzed. The left atrial global longitudinal strain (LA-GLS) was read on the strain curves of AP4 and AP2 respectively (Fig. 1A,B). The longitudinal LA strain curve had a positive systolic peak (SS), an early diastolic plateau (SE), and a late diastolic peak (SA) (Fig. 1C). The longitudinal LA strain rate curve had a positive systolic peak (SRS), an early negative peak at early diastole (SRE), and a late negative peak at late diastole (SRA) (Fig. 1D). SS and SRS represented the LA reservoir function, EDS (= SS − SA) and SRE reflected the LA conduit, and SA and SRA were the indices of the LA booster function. The value was the average of the AP4 and AP2 measurements, presenting as LA longitudinal average strain (LA-mGLS), and average strain rate: LA-mSRS, LA-mSRE and LA-mSRA. All the image and strain analyses were done by a single expert echocardiologist.

### Statistical analysis

The continuous data was demonstrated as means and standard deviations if normally distributed and count data was expressed as a percentage. Chi-square test was used between groups; independent sample T test was used for inter-group test if continuous variables conformed to normal distribution; Variables were entered into multivariate logistic regression analysis and a Pearson test was used for correlation, with P < 0.05 being considered statistically significant. The statistical analyses were done through SPSS 18.0 statistical software.

### Ethics approval and consent to participate

This study was approved by the Ethics Committee of Dalian University, and all participants provided written informed consent.

## Results

### Patients’ characteristics

When comparing the patients’ demographic and clinical data of the CSFP group and control group, there was no statistical difference in age (P = 0.66), gender (P = 0.46), prevalence of hypertension (P = 0.94), diabetes (p = 0.07), systolic blood pressure (P = 0.94), diastolic blood pressure (P = 0.96), heart rate (P = 0.47), and body mass index (P = 0.07), except for smoking history (35.6% in CSFP group vs. 17.2% in control group, P < 0.001) (Table 1).

**Table 1:** Comparison of clinical characteristics between CSF group and control group.

	CSF group (n = 101)	Control group (n = 139)	P
Age (years)	60.84 ± 9.63	60.30 ± 8.91	0.66
Gender (% Male)	55.4 (56)	48.9 (68)	0.46
Smoking history (%)	35.6 (36)	17.2 (24)	0.001
Hypertension (%)	48.2 (67)	53.4 (54)	0.94
Diabetes (%)	20.8 (21)	24.4 (34)	0.07
BMI (kg/m^2^)	25.87 ± 3.22	25.11 ± 3.26	0.07
Systolic BP (mmHg)	137.19 ± 19.07	137.01 ± 16.35	0.94
Diastolic BP (mmHg)	83.33 ± 10.99	83.25 ± 10.85	0.96
Heart rate (beats/min)	68.96 ± 9.90	69.87 ± 9.48	0.47

Values shown are means ± SD or percentages.

*CSF* coronary slow flow, *BMI* Body mass index.

### Coronary angiography and slow blood flow pattern

1,954 consecutive patients underwent coronary angiography due to suspected coronary artery disease during January 2016–September 2017 in the catheterization lab of the affiliated Zhongshan Hospital of Dalian University. Out of those 1954 patients, 512 were included in the study after the exclusion criteria was applied. Based on the recommendations of CSFP by Beltrame et al.^2^, 101 patients were identified to have CSFP (~ 5.5%). At the same time, 139 patients who had normal coronary angiography were selected as the control group from the same data pool. Among 101 patients with CSFP, 50 patients (49.5%) were observed to have all three vessel involvement, 28 patients (27.7%) with two vessel involvement, and 23 patients (22.8%) with only one vessel involvement. The LAD was the most frequently engaged (70.3% of cases), followed by RCA (55.4%) and LCX (46.5%) respectively. Using corrected TIMI frame count (cTFC) to quantify the blood flow velocity, we found that there was a statistical difference between the CSF group and control group, and it was independent of use of different contrast agents (Table 2).

**Table 2:** Coronary angiography and coronary slow flow pattern.

	CSF group (n = 101)	Control group (n = 139)	P
cTFC (LAD)	38.05 ± 18.26	16.99 ± 4.58	< 0.001
cTFC (LCX)	43.37 ± 22.77	18.75 ± 4.48	< 0.001
cIFC (RCA)	46.63 ± 21.87	20.14 ± 4.79	< 0.001
**Types of coronary dominance**			
Right dominant	52.4% (53)	46.7% (65)	0.38
Left dominant	5% (5)	5.7% (8)	0.79
Balanced type	42.6% (43)	47.6% (66)	0.45
**Types of contrast agents**			
Iohexol	83.2% (84)	77.7% (108)	0.21
Iopamidol	16.8% (17)	22.3% (31)	0.19

Values shown are means ± SD or percentages.

*CSF* coronary slow flow, *cTFC* corrected TIMI frame count, *LAD* left anterior descending, *LCX* left circumflex, *RCA* right coronary artery.

### Comparison of laboratory findings in CSF group and Control Group

There was no significant difference between the CSF group and the control group in white blood cell count, red blood cell count, average red blood cell volume, hemoglobin concentration, coefficient of variation of red blood cells, distribution width of red blood cells, platelet count, platelet hematocrit, platelet distribution width, mean platelet volume, proportion of large, albumin, total bilirubin, uric acid, urea nitrogen, fasting blood glucose, creatinine, total triglycerides, high-density lipoprotein, NT-proBNP, homocysteine, and high-sensitivity C-reactive protein. However, a statistically significant difference was observed in the percentage of monocytes, absolute value of monocytes, monocytes cell/high density lipoprotein ratio, hemoglobin, hematocrit, the average erythrocyte hemoglobin concentration, total cholesterol, and low-density lipoprotein (P < 0.05) (Table 3).

**Table 3:** Blood routine and biochemical test information.

	CSF group (n = 101)	Control group (n = 139)	P
WBC (× 10^9^)	5.96 ± 1.38	6.014 ± 1.45	0.77
% of monocytes	4.73 ± 1.80	4.08 ± 1.81	0.007
Monocytes number (× 10^9^)	0.28 ± 0.12	0.25 ± 0.11	0.034
Monocytes/HDL	0.23 ± 0.13	0.19 ± 0.10	0.017
RBC (× 10^12^)	4.62 ± 0.42	4.51 ± 0.43	0.07
Hemoglobin (g/L)	143.18 ± 12.31	139.26 ± 10.26	0.008
I{ematocrit (%)	41.52 ± 3.74	40.18 ± 3.57	0.005
Average RBC vol. (fl)	89.92 ± 3.75	89.06 ± 3.47	0.07
MEH (pg)	30.89 ± 1.99	30.36 ± 1.53	0.02
Hemoglobin Conct. (g/L)	346.03 ± 10.66	344.14 ± 10.19	0.17
Platelet count (× 10^9^)	206.08 ± 46.60	215.84 ± 50.90	0.13
Average platelet vol. (fl)	10.55 ± 0.78	10.60 ± 0.90	0.64
Large platelet ratio(%)	29.33 ± 6.50	29.48 ± 7.59	0.87
Albumin (g/L)	42.36 ± 3.63	42.34 ± 3.39	0.97
Total bilirubin (mmol/L)	13.01 ± 46.42	13.18 ± 5.73	0.82
BG (mmol/L)	5.774 ± 1.57	5.74 ± 1.30	0.86
Urea nitrogen (mmol/L)	5.564 ± 1.40	5.43 ± 1.30	0.44
Creatinine (μmol/L)	65.44 ± 13.84	62.74 ± 13.17	0.13
Uric acid (μmol/L)	348.64 ± 92.76	333.89 ± 83.13	0.20
Triglyceride (mmol/L)	1.81 ± 1.13	1.83 ± 1.25	0.88
TC (mmol/L)	5.23 ± 1.06	4.80 ± 0.96	0.001
HDL (mmol/L)	1.31 ± 0.40	1.33 ± 0.31	0.65
LDL (mmol/L)	2.99 ± 0.84	2.62 ± 0.73	0.001
NT-proBNP (pg/mL)	73.57 ± 86.37	69.21 ± 486.37	0.76
Homocysteine (μmol/L)	11.75 ± 5.63	10.63 ± 4.58	0.46

Values shown are means ± SD or percentages.

*WBC* white blood cells, *RBC* red blood cells, *FBG* fasting blood glucose, *MEH* mean erythrocyte hemoglobin, *TC* total cholesterol, *HDL* high density lipoprotein cholesterol, *LDL* low density lipoprotein cholesterol, *NT-proBNP* N-terminal pro B-type natriuretic peptide.

The univariate variables with statistical differences were then incorporated into the binary logistic regression equation, with the CSF group assigned “1” and the control group assigned “0”. The results showed that the percentage of monocytes was an independent risk factor for the occurrence of coronary artery slow blood flow (OR value 1.354, 95% CI 1.020–1.797, P = 0.036) (Table 4).

**Table 4:** Binary logistic regression analysis.

Variable	B	BE	Wals × 2	OR value	95% CI	P value
Smoking history	0.451	0.382	1.476	0.626	0.294–1.333	0.19
% of monocytes	− 0.304	0.142	4.401	1.354	1.020–1.797	0.036
Monocytes number	3.606	3.412	1.558	3.680	0.046–0.295	0.29
Monocytes/HDI	− 1.467	2.664	0.303	0.231	0.001–0.427	0.58
Hemoglobin	− 0.003	0.020	0.025	0.997	0.958–1.037	0.87
Hematocrit	− 0.102	0.059	2.978	0.903	0.804–1.014	0.08
MEH	− 0.069	0.094	0.534	0.933	0.776–1.123	0.47
Total cholesterol	0.177	0.284	0.390	1.194	0.684–2.083	0.53
LDL cholesterol	0.300	0.366	0.669	1.349	0.658–2.766	0.41

Values shown are means ± SD or percentages.

*MEH* mean erythrocyte hemoglobin, *HDL* high density lipoprotein cholesterol, *LDL* low density lipoprotein cholesterol.

### Echocardiographic findings—left atrial structure and function

There was no statistically significant difference between the CSF group and the control group in the transverse and long diameter of the left atrium, however, there was a statistically significant difference observed in anterior–posterior diameter of the left atrium between the two groups (Table 5).

**Table 5:** Comparison of LA structure of CSF group and control group.

	CSF Group (n = 101)	Control Group (n = 139)	P
LA-ap (mm)	37.81 ± 3.77	36.87 ± 3.51	0.048
LA-t (mm)	38.394 ± 4.45	37.60 ± 3.83	0.15
LA-I (mm)	50.04 ± 4.99	49.00 ± 4.16	0.08
LADI (mm/m^2^)	22.66 ± 2.29	23.09 ± 2.46	0.17

Values shown are means ± SD or percentages.

*LA-ap* anterior–posterior diameter of left atrium, *LA-t* transverse diameter of Ieft atrium, *LA-I* long diameter of left atrium, *LADI* left atrial diameter index.

Using 2D-STE to evaluate the LA function, we observed statistically significant differences regarding the 2D STE-derived indices, including left atrial ejection fraction (LA-EF), left atrium minimum volume index (LAVI-min), left atrium global longitudinal strain (LA-mGLS) and late diastolic strain rate (LA-mSRA) (P < 0.05) (Table 6).

**Table 6:** Comparison of LA 2D-STE in CSF Group and control group.

	CSF group (n = 101)	Control group (n = 139)	P
LA-EF (%)	52.20 ± 9.73	56.31 ± 7.13	0.041
LAVI-max (ml/m^2^)	24.54 ± 4.56	22.78 ± 3.15	0.06
LAVI-min (ml/m^2^)	11.66 ± 2.63	9.84 ± 1.63	0.001
LA-mGLS (%)	28.79 ± 5.67	32.07 ± 5.74	0.016
LA-mSRS (l/s)	0.68 ± 0.33	1.74 ± 0.27	0.44
LA-mSRE (l/s)	− 1.49 ± 0.39	− 1.60 ± 0.31	0.23
LA-mSRA (l/s)	− 1.92 ± 0.33	− 2.10 ± 0.37	0.031

Values shown are means ± SD or percentages.

*LA-EF* left atrial ejection fraction, *LAVI-max* maximum volume index of left atrium, *LAVI-min* minimum volume index of left atrium, *LA-mGLS* average global longitudinal strain of left atrium, *LA-mSRS* left atrial longitudinal strain rate (systolic), *LA-mSRE* left atrial longitudinal strain rate (early diastolic), *LA-mSRA* left atrial longitudinal strain rate (late diastolic).

### Correlation test

**Figure 2:**
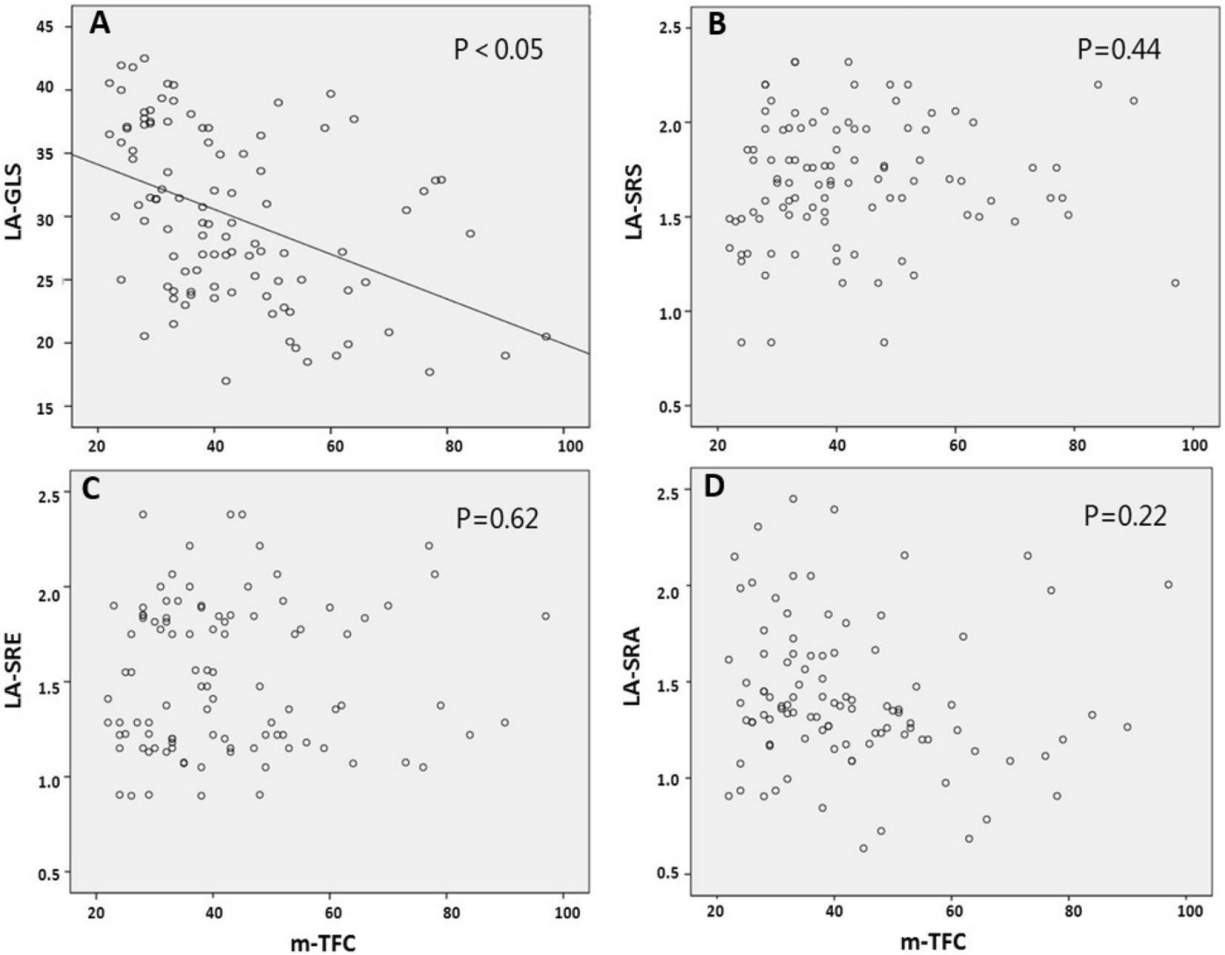
(**A**) LA-GLS and mTFC correlation test; (**B**) LA-SRS and mTFC correlation test; (**C**) LA-SRE and mTFC correlation test; (**D**) LA-SRA and mTFC correlation test. *LA-GLS* left atrial global longitudinal strain, *LA-SRS* left atrial longitudinal strain rate (systolic), *LA-SRE* left atrial longitudinal strain rate (early diastolic), *LA-SRA* left atrial longitudinal strain rate (late diastolic), *mTFC* average of TIMI frame count.

The correlation of continuous variables was selected for analysis by Pearson test, and was aimed at exploring whether the mean blood flow velocity mTFC of the three vessels was correlated with the left atrial strain index. The results showed that there was a negative correlation between LA-GLS and mTFC (P < 0.05), and there was no correlation between LA-SRS, LA-SRE, LA-SRA, and mTFC (P = 0.44; P = 0.62; P = 0.22; Fig. 2).

## Discussion

Unlike the relatively clear definition, the pathophysiology of CSFP is not fully understood. Several possible mechanisms have been proposed, such as the endothelial dysfunction theory^28^, early atherosclerosis^29^, microvascular disease^5,30^, platelet dysfunction^31^, changes in hemorheology^32,33^, and oxidative stress and local/systemic inflammatory response^3–5^. Recently, some studies have indicated that lipoprotein-associated phospholipase A2^34^, plasma choline^35^, or serum salusin beta level^36^ could be a predictive or diagnostic biomarker for CSF.

In this study, we found a statistically significant difference in the percentage of monocytes between the CSF group and the control group. The percentage of monocytes was determined to be an independent risk factor for the occurrence of CSFP after using regression analysis. Monocytes play an important role in the occurrence and development of atherosclerosis, which precipitates the progression of atherosclerotic plaques from a stable state to an unstable state^37,38^. Coronary intravascular ultrasounds have revealed intimal thickening, diffuse vascular wall calcification, and atheromatous plaque formation in the epicardial coronary arteries of CSF patients^38,39^, indicating that CSFP may be an early manifestation of coronary atherosclerosis or part of systemic atherosclerosis^29,38^. Mononuclear cells have been shown to be indirectly involved in three related processes in the development of atherosclerosis^37,38^: (1) mononuclear cells can be activated by risk factors such as smoking, diabetes, hypertension, and hyperlipidemia, which accelerates the progression of atherosclerosis, (2) mononuclear cells participate in the inflammatory response in the acute phase of unstable plaque rupture, acute coronary syndrome, and other homeostatic imbalances, (3) mononuclear cells can enter myocardial tissue in the hypoxic phase during acute coronary events, promoting myofibroblast regeneration, angiogenesis, and repair and remodeling of the myocardium. Although there are studies indicating the involvement of HDL in the regulation of monocyte functions^40^, we did not find a statistical difference in HDL between the CSF group and the control group in our study. In addition, we also noticed a higher prevalence of smoking and a higher BMI in the CSF group than that of the control group, which can be linked with some recent findings. There are several studies indicating that smoking can induce endothelial dysfunction through oxidative stress^41,42^, which is involved in the development of CSFP. Metabolic syndrome (such as obesity, high BMI, diabetes, etc.) has similar effects on vascular endothelium. Obesity and a high BMI can cause endothelial dysfunction through an imbalance in the production of vasodilatory agents, thus inducing CSFP^15,43^.

The left atrium plays an important role in ensuring proper performance of left ventricular function and the systemic circulation. From a hemodynamic perspective, the left atrium possesses three functions: as a reservoir, passive conduit and booster pump^24,44^. The LA size and function has an important clinical and prognosis impact^22,24^. Evaluation of LA size and function can be obtained by traditional 2D-echocardiography, however these parameters are morphometric and static. 2D-STE is a relatively new echocardiographic technique which uses standard B-mode images for regional and global myocardial function analysis. The technology tracks the spatial dislocation of speckles, which is assumed to represent myocardial deformation^44,45^. 2D-STE analysis gives an excellent assessment of the atrial deformation profile during an entire cardiac cycle by closely following the LA physiology^25,46,47^. In contrast to Doppler derived parameters, speckle tracking has the advantages of being angle-independent, less load-dependent than ejection fraction, and less affected by reverberations, side lobes and drop out artifacts. 2D-STE was found to be a feasible, reproducible and sensitive method to assess LA function^47–49^. Several studies have shown that strain imaging can detect LA dysfunction before the manifestation of LA structural changes^49–51^.

The effect of CSFP on LA functions remains controversial^20,51,52^. Wang et al.^52^ evaluated the LA function in 82 patients with CSFP and compared with normal control participants. They observed decreased LA conduit function and increased LA pump function in patients with CSFP, but no significant differences regarding LA phasic emptying volume and emptying fraction were observed between the two groups. However, in another study with 1:1 patient-control matching design for age, sex, hypertension, diabetes mellitus and the LV function by Fallah^20^, the authors did not find significant differences regarding triphasic LA functions assessed through 2D-STE between the CSFP group and control group. In this study, our results showed that minimum left atrial volume index (LAVImin) increased and left atrial ejection fraction (LA-EF) decreased in the CSFP group compared with the control group, although no statistically significant differences were found in the LA size (i.e., antero-posterior diameter, lateral diameter and LA length) and maximum left atrium volume index (LAVIm) between the two groups. Meanwhile, we found that patients with CSF have lower LA-mLGS and higher late diastolic strain rate (LA-mSRA), suggesting an impairment of left atrial reservoir function and booster function in patients with CSFP. The correlation test revealed a negative correlation between LA-mGLS and mTFC, suggesting that the overall LA function will further decrease with a decrease in coronary blood flow velocity.

### Study limitations

Firstly, this is a single-center conducted retrospective study and 2D-STE data was measured by a single-skilled echocardiologist. Secondly, the patients’ usage of medications was not taken into consideration. Finally, no follow-up data was provided at this time.

## Conclusion

In conclusion, the CSFP was relatively frequent. The percentage of mononuclear cells can be considered independent predictors of this phenomenon. The functions of the LA (i.e., reservoir and booster pump) were impaired in patients with the CSFP as evaluated through 2D-STE.

## Data Availability

The datasets used and/or analyzed in the study are available from the corresponding author upon reasonable request.

## Abbreviations

CSFP: Coronary slow flow phenomenon
LA: Left atrium
TIMI: Thrombolysis in myocardial infarction
2D-STE: Two-dimensional speckle tracking echocardiography
cTFC: Corrected TIMI frame count
WBC: White blood cells
RBC: Red blood cells
FBG: Fasting blood glucose
MEH: Mean erythrocyte hemoglobin
TC: Total cholesterol
HDL: High density lipoprotein cholesterol
LDL: Low density lipoprotein cholesterol
LAD: Left anterior descending
LCX: Left circumflex
RCA: Right coronary artery
LA-ap: Left atrial anterior–posterior diameter
LA-t: Left atrial transverse diameter
LA –l: Left atrial long diameter
LADI: Left atrial diameter index
LA-EF: Left atrial ejection fraction
LAVI-max: Left atrial maximum volume index
LAVI-min: Left atrial minimum volume index
SS: Systolic longitudinal strain
SE: Early diastolic strain
SA: Late diastolic strain
LA-GLS: Left atrial global longitudinal strain
LA-SRS: Left atrial longitudinal strain rate (systolic)
LA-SRE: Left atrial longitudinal strain rate (early diastolic)
LA-SRA: Left atrial longitudinal strain rate (late diastolic)
